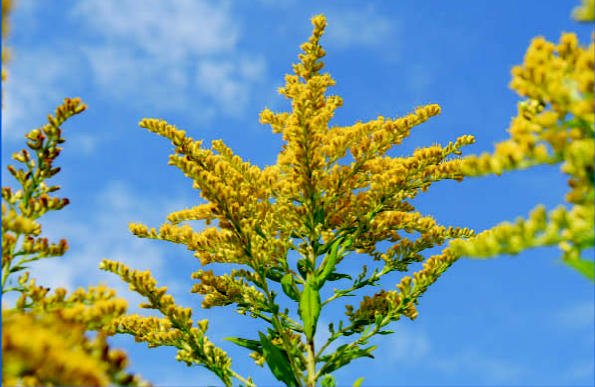# Headliners: Allergies: Ragweed Subpollen Particles Reach Deep into Lungs

**DOI:** 10.1289/ehp.115-a193

**Published:** 2007-04

**Authors:** Jerry Phelps

Bacsi A, Choudhury BK, Dharajiya N, Sur S, Boldogh I. 2006. Subpollen particles: carriers of allergenic proteins and oxidases. J Allergy Clin Immunol 118:844–850.

During the flowering season, high humidity and moisture trigger the release of pollen grains from grasses, trees, and shrubs. Allergens contained in these pollen grains can cause reactions in the skin, eyes, and upper and lower respiratory tracts. Seasonal asthma also is associated with pollen exposure.

How pollen allergens contribute to inflammation in the lower airways has puzzled researchers since few pollen grains reach the peripheral airways due to their size. In this report, NIEHS grantees Sanjiv Sur and Istvan Boldogh, with colleagues at the University of Texas Medical Branch in Galveston, present new findings suggesting that fragments of pollen grains, called sub-pollen particles (SPPs), are capable of reaching the lower airway regions and causing the clinical symptoms associated with seasonal asthma.

This same research team recently reported that ragweed pollen grains contain intrinsic NAD(P)H oxidases, and that exposure to them generates oxidative stress in the airway epithelium within minutes of exposure. In the current study, the investigators analyzed bronchial epithelial cells after exposure to hydrated short ragweed (*Ambrosia artemisiifolia*) and redroot pigweed (*Amaranthus retroflexus*) pollen grains. They also used an experimental mouse model of asthma to challenge sensitized mice with intranasally applied SPPs.

The researchers found that ragweed pollen grains release SPPs in the size range of 0.5–4.5 μm, small enough to reach lower airways. They determined that the SPPs contained allergenic proteins and possessed NADH or NAD(P)H oxidase activity. Exposure of cultured cells to SPPs caused significant increases in the generation of reactive oxygen species and induced airway inflammation in laboratory mice. Pretreatment of the SPPs with NADH and NAD(P)H oxidase inhibitors reduced their ability to increase reactive oxygen species in the airway epithelial cells and reduced airway inflammation.

This is the first report to demonstrate the presence of allergenic proteins and oxidase activity in SPPs of respirable size. The study provides insight into the potential role of SPPs in seasonal asthma and suggests that oxidase inhibitors may be useful therapeutic agents in reducing or preventing oxidative damage and inflammation.

## Figures and Tables

**Figure:**